# Quantitative emphysema on computed tomography imaging of chest is a risk factor for prognosis of esophagectomy: A retrospective cohort study

**DOI:** 10.1097/MD.0000000000035547

**Published:** 2023-10-13

**Authors:** Hiroki Mizusawa, Osamu Shiraishi, Masashi Shiraishi, Ryuji Sugiya, Tamotsu Kimura, Akira Ishikawa, Takushi Yasuda, Yuji Higashimoto

**Affiliations:** a Faculty of Medicine, Department of Rehabilitation Medicine, Kindai University, Osaka, Japan; b Department of Public Health, Graduate School of Health Sciences, Kobe University, Kobe, Japan; c Faculty of Medicine, Department of Surgery, Kindai University, Osaka, Japan; d Department of Respiratory Medicine and Allergology, School of Medicine, Kindai University, Osaka, Japan.

**Keywords:** comorbidity, emphysema, esophagectomy, low attenuation area, smoking

## Abstract

The low attenuation area percentage (LAA%) is gaining popularity. LAA% is an index of quantitative emphysema on computed tomography (CT) imaging of the chest. This study aims to retrospectively investigate whether preoperative LAA% is associated with postoperative prognosis in patients with esophageal cancer who were scheduled for esophagectomy. From January 2016 to March 2020, 105 patients with esophageal cancer underwent esophagectomy via right thoracotomy and neoadjuvant chemotherapy. A Synapse Vincent volume analyzer (Fujifilm Medical, Tokyo, Japan) was used for measurement. The software automatically quantified LAA% using a threshold of less than − 950 Hounsfield units on CT images of lung regions. Cox proportional hazard analyses were performed in univariable and multivariable forms. Estimates of the receiver operating curve are used to determine the cutoff value for death of LAA%, and the binary value is then inserted into Cox proportional hazard analyses. The preoperative LAA% cutoff value was ≥ 6.3%. Patients with a preoperative LAA% ≥6.3% had a significantly worse prognosis than those with a preoperative LAA% of < 6.3%. LAA% ≥6.3% (hazard ratio: 6.76; 95% confidence interval: 2.56–17.90, *P* < .001) was the most influential preoperative factor for overall survival after esophagectomy in multivariate Cox proportional hazard analyses. LAA% is one of the preoperative risk factors for survival after esophagectomy and an indicator of lung condition using routinely performed preoperative CT images. We quantified the extent of preoperative emphysema in patients with esophageal cancer, who were scheduled for surgery, and for the first time, reported LAA% as one of the preoperative risk factors for survival after esophagectomy.

## 1. Introduction

Esophageal cancer is the 8th most common cancer in the world.^[1]^ The most invasive thoracic surgery is esophagectomy, and surgery plays a significant role in achieving locoregional control and offers the best chance of local and advanced disease treatment in esophageal cancer.^[2,3]^ In Europe and America, the overall survival (OS) of esophagectomy via open thoracotomy is approximately 30% to 60% over the last decade.^[4]^ Chemotherapy, a widely used neoadjuvant chemotherapy (NAC), is indicated to improve the postoperative prognosis of esophagectomy.^[5–7]^ Docetaxel, cisplatin, and 5-fluorouracil or 5-fluorouracil, docetaxel, and nedaplatin (UDON) are the main types of chemotherapy that are used in NAC in Japan.^[8,9]^ Clinical tumor, nodal, metastasis stage is not the only factor that influences esophagectomy prognosis; individual patient comorbidities are also important factors in postoperative prognosis.^[10,11]^ Smoking history and comorbid chronic obstructive pulmonary disease (COPD) are risk factors for postoperative pneumonia after esophagectomy, and postoperative pneumonia has an impact on postoperative prognosis.^[12,13]^ Smoking is one of the risk factors for esophageal squamous cell carcinoma (SCC) of esophageal cancer.^[1]^ Given this, it is speculated that many patients with esophageal cancer have poor lung conditions due to smoking exposure.

It is important to know the preoperative lung condition in esophagectomy, and many facilities perform preoperative spirometry.^[14]^ However, spirometry may impair objectivity because the results are influenced by the examinee’s cognitive function and the examiner’s skill.^[15,16]^ Multi-detection using computed tomography (CT) image data and image analysis using artificial intelligence have been widely used in clinical settings in recent years.^[17]^ Among these, the low attenuation area percentage (LAA%), first reported by Mishima et al^[18]^ in 1999. LAA% is a quantitative emphysema index based on CT imaging of the chest and a percentage of total lung volume.^[18,19]^ LAA% is related to forced expiratory volume in 1 second (FEV1)/forced vital capacity (FVC) and lung carbon monoxide diffusing capacity.^[20]^ Individuals with a smoking history who were either currently smoking or had smoked in the past had a 1.0% increase in LAA% that was independently associated with all-cause mortality.^[21]^ Although spirometry is not associated with prognosis in COPD patients, there is an association between LAA% and prognosis.^[22]^ Preoperative LAA% before lung resection in patients with lung cancer is reported to be associated with postoperative respiratory complications and postoperative prognosis.^[23,24]^ In light of these considerations, LAA%, which quantifies pulmonary emphysema in patients with esophageal cancer who were scheduled for surgery, may have an impact on postoperative prognosis.

This study aims to retrospectively investigate whether preoperative LAA% is associated with postoperative prognosis in patients with esophageal cancer who were scheduled for esophagectomy.

## 2. Materials and methods

### 2.1. Patients and study design

This study is a single center, retrospective cohort study. Patients with thoracic esophageal cancer who underwent esophagectomy via right thoracotomy and NAC at Kindai University Hospital between January 2016 and March 2020 were included in the study. Consecutive patients during this period were also enrolled. The exclusion criteria were as follows: Secondary surgery; With residual tumor, and; The LAA% could not be measured. This study was approved by the Committee for Ethics at Kindai University School of Medicine (no. R02–115), and it was conducted in accordance with the ethical standards established in the 1964 Declaration of Helsinki and subsequent amendments. Regarding informed consent, information on this study was disclosed on our hospital website, and opt-out was used. Regarding informed consent, information on this study was disclosed on our hospital website, and opt-out was used. No one offered to refuse study participation. This opt-out procedure was approved by the Committee for Ethics at Kindai University School of Medicine.

### 2.2. LAA% analysis

Emphysema was evaluated using CT with a 5.0-mm slice taken a month before esophagectomy. A Synapse Vincent volume analyzer (Fujifilm Medical, Tokyo, Japan) was used to measure the LAA%. The software automatically quantified LAA% in bilateral lung areas using threshold values of less than − 950 Hounsfield units (HU) to distinguish emphysema from other tissues.^[21]^ We calculated the LAA% by subtracting the associated emphysema volume from the total lung volume and applying the lower threshold value of − 950 HU to the percentage of the whole lung.

### 2.3. Sample size

The less eventful group required a minimum of 10 cases for univariate analysis of regression in Cox proportional hazards analysis. A previous study reported at least approximately 70% 5-year survival rate for esophagectomy.^[4]^ Hence, assuming that the event occurrence is death, this study required 34 patients, which is the minimum sample size required.

### 2.4. Statistical analysis

Data on the continuous variables are expressed as the median (interquartile range [IQR]). All statistical analyses were performed with EZR ver1.41 (Saitama Medical Center, Jichi Medical University, Saitama, Japan).^[25]^ The hazard ratio (HR) of clinical parameters on OS after esophagectomy was investigated using univariable and multivariable Cox proportional hazard analyses. In the Cox proportional hazard analyses, clinical parameters included age, gender, body mass index, clinical tumor, nodal, metastasis stage, and response to neoadjuvant therapy according to Japanese Society for Esophageal Disease criteria,^[26]^ FEV1/FVC ratio, Charlson comorbidity index (CCI) of total score of weighted comorbidities, and LAA%. Estimates of the receiver operating curve are used to determine the cutoff value for death of LAA%, and the binary value is then inserted into Cox proportional hazard analyses. Clinically relevant factors with *P* values < .1 in a Cox proportional hazard model with univariable analysis were considered potential risk factors and were further investigated using a multivariable Cox hazard model. The results of the Cox proportional hazards analysis are shown as HRs with 95% confidence intervals (95% CIs). *P* values < .05 were deemed statistically significant.

## 3. Results

### 3.1. Patient characteristics

**Figure 1.**
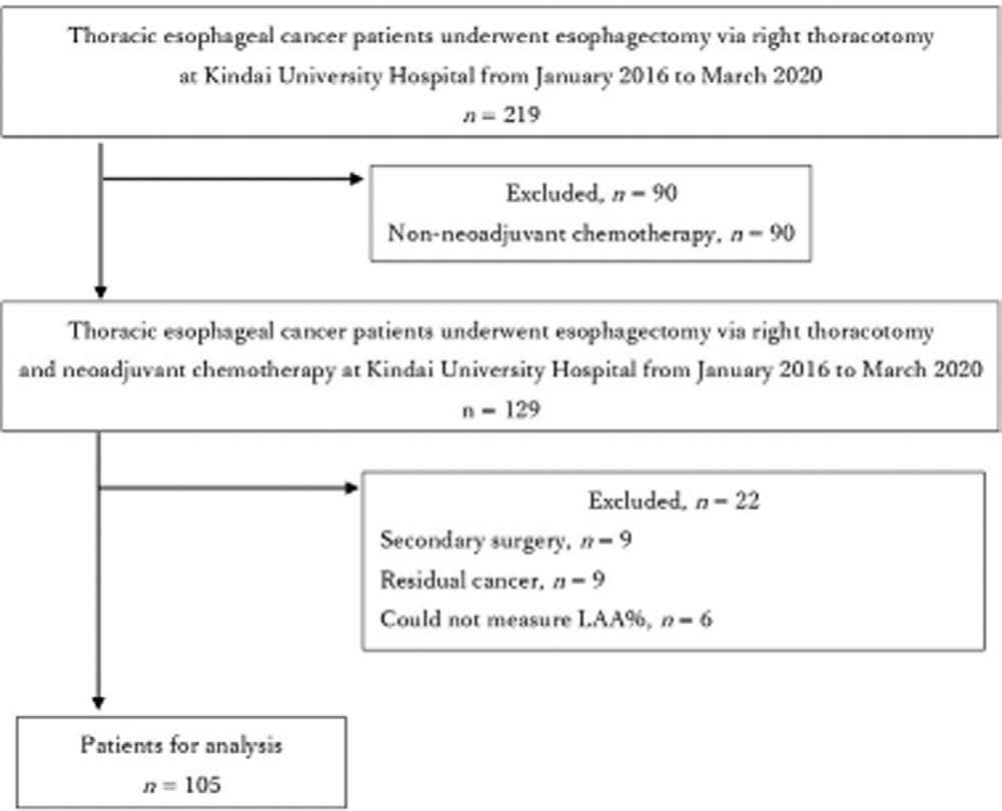
Flowchart depicting the patient selection process.

**Figure 2.**
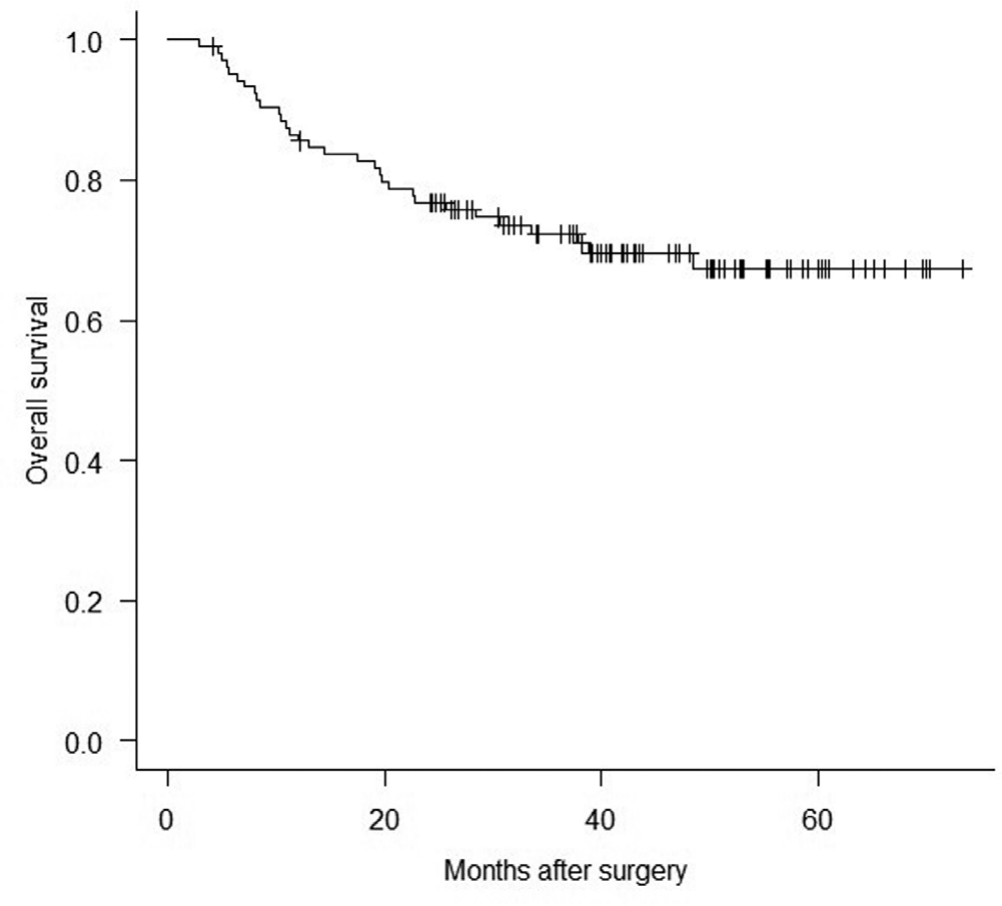
The overall survival (OS) curves were constructed using the Kaplan–Meier method in all patients. The 5-yr OS rate is 67.3%, and the median follow-up period is 43.8 months (interquartile range, 35.2–56.2 months).

**Figure 3.**
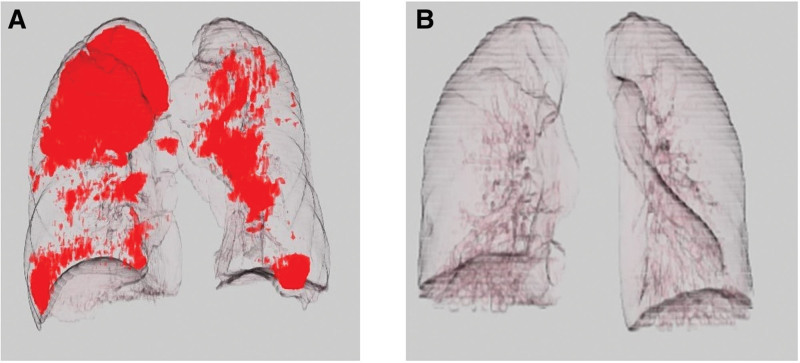
(A) Highest low attenuation area percentage (LAA%) in this study participants, with a 13.1%. (B) Lowest LAA%, which is 0.0%. Areas shown in red are below − 950 Hounsfield units (HU), indicating emphysema in the lung.

**Figure 4.**
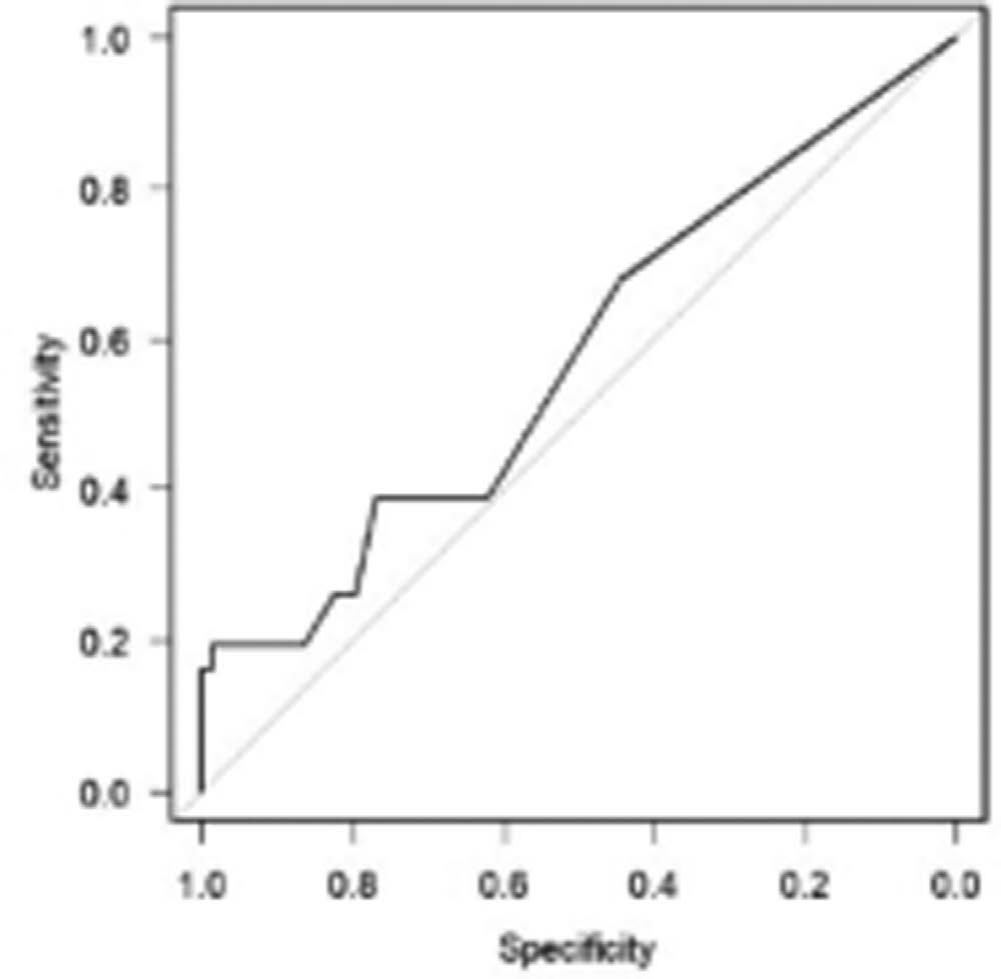
Receiver operating characteristics (ROC) curves of low attenuation area percentage (LAA%) on logistic regression analysis were constructed in the study participants. Area under the curve of LAA% was 0.576, and the cutoff value was 6.3% (specificity: 0.986, sensitivity: 0.194).

Between January 2016 and March 2020, 219 patients had esophagectomy and reconstruction, with 90 having no NAC and the other 129 having NAC. Nine of the 129 patients had secondary surgery, 9 had residual tumor, and 6 patients were unable to measure LAA% accurately because the software recognized the stomach, intestines, and trachea as part of the lungs in measuring LAA%. After the 24 patients were excluded, 105 patients were eligible for analysis (Fig. 1). Table 1 summarizes the backgrounds of all 105 patients studied. Eighty-three males (79.1%), median age of 69.0 years (IQR: 62.0–73.0 years), 87 (82.9%) former smokers, 94 (89.5%) SCC, 65 (61.9%) clinical stage III or higher, 73 (69.5) had NAC docetaxel/cisplatin/5-fluorouracil, 73 (69.5%) had NAC response complete response/partial response, and the median FEV1/FVC ratio was 74.6% (IQR: 70.5%–79.5%). Figure 2 depicts the overall patient survival rate, with 31 (29.5%) dying from any cause and 5-yr survival rate of 67.3%. Esophageal cancer was responsible for 21 (20.0%) of the deaths, whereas other diseases (5 patients died of pneumonia) were responsible for 10 (9.5%). The median follow-up period is 43.8 months (IQR: 35.2–56.2 months). Prognosis could not be completely followed in 4 of 105 (3.8%) patients. The preoperative LAA% median was 0.1% (IQR: 0.0%–0.4%; range: 0.0%–13.1%, Fig. 3). The preoperative LAA% cutoff value for dead after esophagectomy was ≥ 6.3% (specificity: 0.986, sensitivity: 0.194; Fig. 4). There were 7 patients with esophageal cancer with a preoperative LAA% ≥6.3%. Six of the 7 patients died, 4 of whom died due to cancer. Patients with a preoperative LAA% of ≥ 6.3% had a significantly worse prognosis than those with a preoperative LAA% of < 6.3% (*P* = .028, did not shown figure or table). The study results revealed that patients with esophageal cancer with LAA% of ≥ 6.3% tended to have a higher percentage of dose reduction with NAC (*P* = .07) (Table S1, Supplemental Digital Content, http://links.lww.com/MD/K231).

**Table 1:** Baseline characteristics of all patients in the study.

	All patients (n = 105)
Age (yr)	69.0 (62.0–73.0)
Male, *n* (%)	83 (79.1%)
BMI (kg/m^2^)	20.4 (18.3–22.7)
Former smokers, *n* (%)	87 (82.9%)
Smoking intensity (cigarettes, day)	20.0 (10.0–30.0)
Smoking duration (yr)	38.0 (15.0–42.5)
Tumor site (upper/middle/lower)	23/41/41
Tumor type (SCC/AC/others)	94/7/4
cStage (I/II/III/IV)	6/34/56/9
cT (1/2/3/4)	7/26/63/9
cN (0/1/2/3)	21/49/34/1
cM (0/1)	92/13
NAC (DCF/others)	73/32
Response to NAC (CR/PR/SD/PD)	3/70/22/10
VC (%)	100.0 (92.0–107.9)
FVC (%)	101.0 (92.4–110.5)
FEV_1_ (%)	92.0 (81.3–101.0)
FEV1/FVC (%)	74.6 (70.5–79.5)
LAA (%)	0.1 (0.0–0.4)
CCI	0.0 (0.0–2.0)
COPD, *n* (%)	5 (4.8%)

Continuous variables are presented as the median (interquartile range [IQR]) and categorical variables by *n* (%).

AC = adenocarcinoma, BMI = body mass index, CCI = Charlson comorbidity index, cM stage = clinical metastasis stage, cN stage = clinical nodal stage, COPD = chronic obstruction pulmonary disease, CR = complete response, cStage = clinical stage, cT stage = clinical tumor invasion stage, DCF = docetaxel/cisplatin/5-fluorouracil, FEV1 = forced expiratory volume in a second, FVC = forced vital capacity, LAA% = low attenuation area percentage, NAC = neoadjuvant chemotherapy, PD = progressive disease, PR = partial response, SCC = squamous cell carcinoma, SD = stable disease, VC = vital capacity.

### 3.2. Cox proportional hazard analysis

Using univariable and multivariable Cox proportional hazard analyses, the effect of each clinical parameter on OS after esophagectomy was evaluated (Table 2). In the univariable analyses, the factors of *P* < .1 were age ≥ 65 (HR: 2.56; 95% CI: 1.05–6.24, *P* = .039), clinical nodal stages 1 to 3 (HR: 4.30; 95% CI: 1.05–18.42, *P* = .043), CCI of ≥ 2 (HR: 4.83; 95% CI: 2.37–9.81, *P* < .001), NAC response stable disease/progressive disease (HR: 2.68; 95% CI: 1.32–5.43, *P* = .006), and LAA% of ≥ 6.3% (HR: 5.70; 95% CI: 2.30–14.12, *P* < .001). Multivariate Cox proportional hazard analyses were performed to compare the contributions of these variables. CCI of ≥ 2 (HR: 4.02; 95% CI: 1.92–8.41, *P* < .001), NAC response stable disease/progressive disease (HR: 2.31; 95% CI: 1.12–4.80, *P* = .024), and LAA% of ≥ 6.3% (HR: 6.76; 95% CI: 2.56–17.90, *P* < .001) were significantly associated with a lower risk of death after esophagectomy.

**Table 2:** The univariable and multivariable analysis results of Cox proportional hazard regression models on overall survival (OS) in patients who underwent esophagectomy.

	Univariable analysis			Multivariable analysis		
	HRs	95% CIs	*P* value	HRs	95% CIs	*P* value
Age						
<65 (ref)	–	–	–	–	–	–
≥65	2.56	1.05–6.24	.039*	2.23	0.86–5.77	.099
Gender						
Female (ref)	–	–	–	–	–	–
Male	1.80	0.63–5.16	.272	†	†	†
cT stage						
1–2 (ref)	–	–	–	–	–	–
3–4	0.99	0.47–2.10	.979	†	†	†
cN stage						
0 (ref)	–	–	–	–	–	–
1–3	4.30	1.05–8.42	.043*	2.24	0.50–9.97	.290
cM stage						
0 (ref)	–	–	–	–	–	–
1	0.82	0.25–2.71	.749	†	†	†
BMI						
>18.5 (ref)	–	–	–	–	–	–
≤18.5	1.32	0.62–2.80	.476	†	†	†
CCI						
<2 (ref)	–	–	–	–	–	–
≥2	4.83	2.37–9.81	<.001*	4.02	1.92–8.41	<.001*
FEV1/FVC						
≥70.0 (ref)	–	–	–	–	–	–
<70.0	1.07	0.46–2.48	.879	†	†	†
NAC response						
CR/PR (ref)	–	–	–	–	–	–
SD/PD	2.68	1.32–5.43	.006*	2.31	1.12–4.80	.024*
LAA%						
<6.3% (ref)	–	–	–	–	–	–
≥6.3%	5.70	2.30–14.12	<.0001*	6.76	2.56–17.90	<.001*

BMI = body mass index, CCI = Charlson comorbidity index, cM stage = clinical metastasis stage, cN stage = clinical nodal stage, CR = complete response, cT stage = clinical tumor invasion stage, FEV1 = forced expiratory volume in a second, FVC = forced vital capacity, HR = hazard ratio, LAA% = low attenuation areapercentage, NAC = neoadjuvant chemotherapy, PD = progressive disease, ref = reference, SD = stable disease, VC = vital capacity.

* *P *< .05 (statistically significant).

† Not included in analysis.

## 4. Discussion

In this study, we first applied LAA%, a preoperative CT image-based index, to patients with esophageal cancer who were said to have poor lung conditions due to smoking exposure. The results showed that preoperative LAA% was significantly associated with postoperative prognosis. The preoperative LAA% cutoff value for the postoperative mortality rate in this study’s esophageal cancer patients was 6.3%. Patients with esophageal cancer with a preoperative LAA% ≥6.3% had a high mortality rate and a poor prognosis due to primary disease. The fact that preoperative LAA% was one of the risk factors for postoperative prognosis in our subjects, many of who had esophageal cancer were exposed to smoking, provides new information for esophageal cancer patients scheduled for surgery.

LAA% is an index that quantifies emphysema in the lung by artificial intelligence analysis of chest CT images, identifying areas below a certain HU as emphysema. The activation of macrophages and increased production of CD8^+^ T cells caused by smoking exposure and destruction of alveolar walls caused by increased resistance to corticosteroids are the mechanisms of pulmonary emphysema.^[27]^ Therefore, pulmonary emphysema also develops in healthy smokers without a diagnosis of COPD, and every 1.0% increase in LAA% is associated with a significant increase in all-cause mortality.^[21]^ The higher the LAA% in patients with COPD, the worse the prognosis, with the highest mortality due to respiratory failure.^[22]^ This suggests that the higher the LAA%, the worse the lung condition, and that the LAA% is a prognostic indicator for patients with esophageal cancer and other diseases.

The study results revealed that patients with esophageal cancer with LAA% of ≥ 6.3% tended to have a higher percentage of dose reduction with NAC. Currently, neoadjuvant therapy is considered a standard treatment for patients with stage ≥ II esophageal cancer.^[28,29]^ Dose reductions are generally practiced when chemotherapy causes severe adverse events.^[30]^ Surgery is the primary curative therapy for esophageal cancer, and NAC is a supportive therapy. Therefore, elderly patients or those with renal dysfunction or frailty may be administered a reduced dose of NAC, although patients with esophageal cancer who receive reduced NAC doses have a poor prognosis after esophagectomy.^[31]^ Therefore, patients with esophageal cancer who have a high degree of emphysema might have a poor prognosis due to dose reduction, considering NAC-induced adverse events.

SCC accounts for a large proportion of esophageal cancer in Asian and African countries, while adenocarcinoma recently accounts for a large proportion of esophageal cancer in Western countries.^[1]^ Tabaco use and alcohol consumption are the most common risk factors for SCC, while symptomatic gastro-esophageal reflux disease, Barrett esophagus, obesity, and tobacco use are risk factors for adenocarcinoma.^[1]^ The influence of tobacco use in adenocarcinoma is not as great as that in Barrett esophagus or obesity, but it is one of the risk factors for adenocarcinoma development.^[32]^ Therefore, we believe that LAA% is clinically useful in prognostic risk stratification before esophagectomy even in Western countries. However, LAA% is only clinically applicable to facilities that have CT scans and dedicated software to measure LAA%, thus some facilities cannot measure LAA% in certain practice settings.

This study had some limitations. First, this study was conducted at a single center with a small number of patients. So, to standardize the patient’s background before esophagectomy, we only included patients who were receiving NAC in our study. Second, there were insufficient independent variables related to esophageal cancer survival. However, it is important to predict the postoperative prognosis based on information obtained before surgery. Third, patients with high LAA% have a higher risk of lung cancer incidence.^[33,34]^ Lung cancer is considered a disease associated with emphysema other than COPD, but none of the patients analyzed in this study had lung cancer before esophagectomy. This study does not discuss the relationship between LAA% and diseases other than COPD because no previous studies have reported that diseases other than COPD affect the degree of emphysema.

## 5. Conclusion

In this study, we discovered that LAA%, a lung condition indicator derived from routinely performed preoperative CT images, is one of the risk factors for survival after esophagectomy. A preoperative LAA% cutoff value ≥ 6.3% should be recognized as an important preoperative index that influences the postoperative prognosis of patients with esophageal cancer scheduled for surgery Preoperative LAA%, with further validation, may be clinically useful to stratify risk in patients who were scheduled for esophagectomy.

## Author contributions

**Conceptualization:** Hiroki Mizusawa.

**Data curation:** Hiroki Mizusawa, Osamu Shiraishi.

**Formal analysis:** Hiroki Mizusawa.

**Investigation:** Hiroki Mizusawa.

**Methodology:** Hiroki Mizusawa.

**Project administration:** Hiroki Mizusawa, Tamotsu Kimura, Akira Ishikawa, Takushi Yasuda.

**Resources:** Hiroki Mizusawa, Osamu Shiraishi.

**Software:** Hiroki Mizusawa.

**Supervision:** Osamu Shiraishi, Masashi Shiraishi, Ryuji Sugiya, Tamotsu Kimura, Akira Ishikawa, Takushi Yasuda, Yuji Higashimoto.

**Validation:** Osamu Shiraishi, Akira Ishikawa, Takushi Yasuda, Yuji Higashimoto.

**Writing – original draft:** Hiroki Mizusawa.

**Writing – review & editing:** Osamu Shiraishi, Yuji Higashimoto.

## Supplementary Material
