# Supplementary material for: Quantitative emphysema on computed tomography imaging of chest is a risk factor for prognosis of esophagectomy: A retrospective cohort study
Source: Medicine (Baltimore). 2023 Oct 13;102(41):e35547. doi: 10.1097/MD.0000000000035547 (PMC10578713; doi:10.1097/MD.0000000000035547)
Supplement: Supplementary file 1 [file medi-102-e35547-s001.docx]

Supplemental Table 1

Comparison of the percentage of patients with dose reduction (<80% RDI) in NAC in the group below the LAA% cutoff and in the group above the cutoff.

|  | LAA% < 6.3% (n = 98) | LAA% ≥ 6.3% (n = 7) | *p* value |
| --- | --- | --- | --- |
| < 80% RDI, n (%) | 23 (23.5%) | 4 (57.1%) | .07 |

LAA%: Low attenuation area percentage, RDI: Relative dose intensity
